# Genomic and Metabolomic Insights into the Natural Product Biosynthetic Diversity of a Feral-Hog-Associated *Brevibacillus laterosporus* Strain

**DOI:** 10.1371/journal.pone.0090124

**Published:** 2014-03-03

**Authors:** Christine M. Theodore, Blake W. Stamps, Jarrod B. King, Lauren S. L. Price, Douglas R. Powell, Bradley S. Stevenson, Robert H. Cichewicz

**Affiliations:** 1 Department of Chemistry and Biochemistry, University of Oklahoma, Norman, Oklahoma, United States of America; 2 Natural Products Discovery Group, Institute for Natural Products Applications and Research Technologies, University of Oklahoma, Norman, Oklahoma, United States of America; 3 Department of Microbiology and Plant Biology, University of Oklahoma, Norman, Oklahoma, United States of America; University of New South Wales, Australia

## Abstract

Bacteria associated with mammals are a rich source of microbial biodiversity; however, little is known concerning the abilities of these microbes to generate secondary metabolites. This report focuses on a bacterium isolated from the ear of a feral hog from southwestern Oklahoma, USA. The bacterium was identified as a new strain (PE36) of *Brevibacillus latersporus*, which was shown *via* genomic analysis to contain a large number of gene clusters presumably involved in secondary metabolite biosynthesis. A scale-up culture of *B. latersporus* PE36 yielded three bioactive compounds that inhibited the growth of methicillin-resistant *Staphylococcus aureus* (basiliskamides A and B and 12-methyltetradecanoic acid). Further studies of the isolate's secondary metabolome provided both new (auripyrazine) and previously-described pyrazine-containing compounds. In addition, a new peptidic natural product (auriporcine) was purified that was determined to be composed of a polyketide unit, two L-proline residues, two D-leucine residues, one L-leucine residue, and a reduced L-phenylalanine (L-phenylalanol). An examination of the genome revealed two gene clusters that are likely responsible for generating the basiliskamides and auriporcine. These combined genomic and chemical studies confirm that new and unusual secondary metabolites can be obtained from the bacterial associates of wild mammals.

## Introduction

Nature has served as a valuable source of bioactive compounds with many natural products (secondary metabolites) having entered into clinical use [1]. The sustained successful application of microbes, plants, and marine life for the identification of new and inspiring secondary metabolites is a testament to their immense biological and chemical diversity [1]. Bioactive substances with unique chemical features have been discovered from a multitude of organisms inhabiting terrestrial and marine environments. In order to maintain a rich pipeline for secondary metabolite discovery, researchers must continue to direct efforts toward exploring previously unexploited biological resources [2].

The microbial associates of animals, especially those from vertebrate hosts, represent a virtually untapped source of bacterial and archaeal diversity [3], [4]. These microorganisms participate in a range of transient and long-term (i.e., symbiotic) relationships with animal hosts [5]. The spectrum of habitats afforded by the abundance of discrete microenvironments in and on a mammal's body substantially increases the variety of microbial species that can inhabit a single animal [6], [7], [8]. Bacteria associated with other microorganisms, plants, nematodes, insects and sponges produce an intriguing variety of secondary metabolites [9], [10]; however, relatively little is known about the natural products generated by the microbes associated with wild mammals. In contrast, mammals such as humans and domesticated animals host a large microbial population with some members engaged in the production of secondary metabolites [11]. Secondary metabolites isolated from microbes associated with the human body have been shown to exhibit antibiotic [12], cytotoxic [13], anti-biofilm [14], [15], and anti-tumor [16] properties. Therefore, it is reasonable to expect that bacteria associated with wild mammals will also be capable of generating secondary metabolites.

This report describes the use of an opportunistic sampling approach [17] to access secondary metabolites produced by a bacterium obtained from the ear canal of a wild mammal. A new, natural-product-producing strain of *Brevibacillus laterosporus* was obtained from a feral hog originating in southwestern Oklahoma, USA. The natural product biosynthetic potential of this isolate was revealed using a combination of LC-MS, bioassays, and genomic data. These efforts provided several compounds including a new and unusual peptidic metabolite, auriporcine (**6**); a new pyrazine, auripyrzine (**5**); and the previously described antifungal metabolites basiliskamides A and B (**1** and **2**, respectively). This research highlights how the integrated application of genomics and metabolomics presents an opportunity for mining new natural products from bacteria associated with wild mammals.

## Results and Discussion

**Figure 1:**
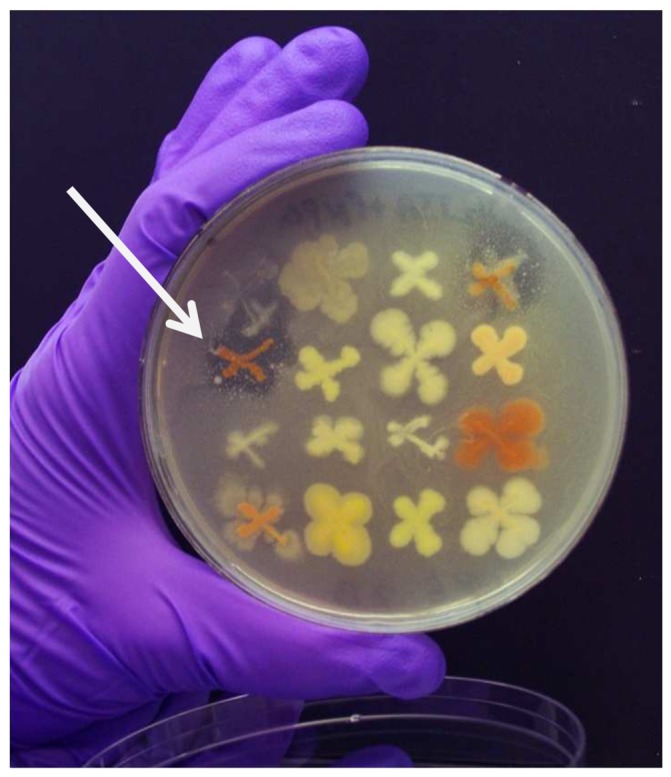
Antimicrobial overlay plate for detecting organisms from the feral hog ear that inhibited *S. aureus* growth. Bacterial isolates were streaked in “X” shaped patterns onto half-strength TSA agar. The *B. laterosporus* PE36 isolate used in this investigation is indicated by the arrow.

Using an opportunistic sampling approach [17], the oral cavity, ear canal, and nasal cavity of a feral hog taken by a hunter in southwestern Oklahoma were swabbed for microbial inhabitants within 24 h of being bagged. The samples were spread onto agar-based media and over 160 bacterial colonies were streaked onto fresh plates. Isolates exhibiting homogenous morphologies were arrayed onto new plates, incubated for several days, and agar overlays seeded with methicillin-resistant *Staphylococcus aureus* were applied over the surfaces of the plates. A number of isolates exhibited antibiosis toward *S. aureus* as demonstrated by zones of inhibition devoid of visible growth of the pathogen in the overlay layer (Figure 1). Several of the active bacteria from the hog's ear exhibited the same phenotypic characteristics (small to medium colony size with dark yellow-orange pigmentation) (Figure 1) and one representative isolate (PE36) was selected for further investigation.

**Figure 2:**
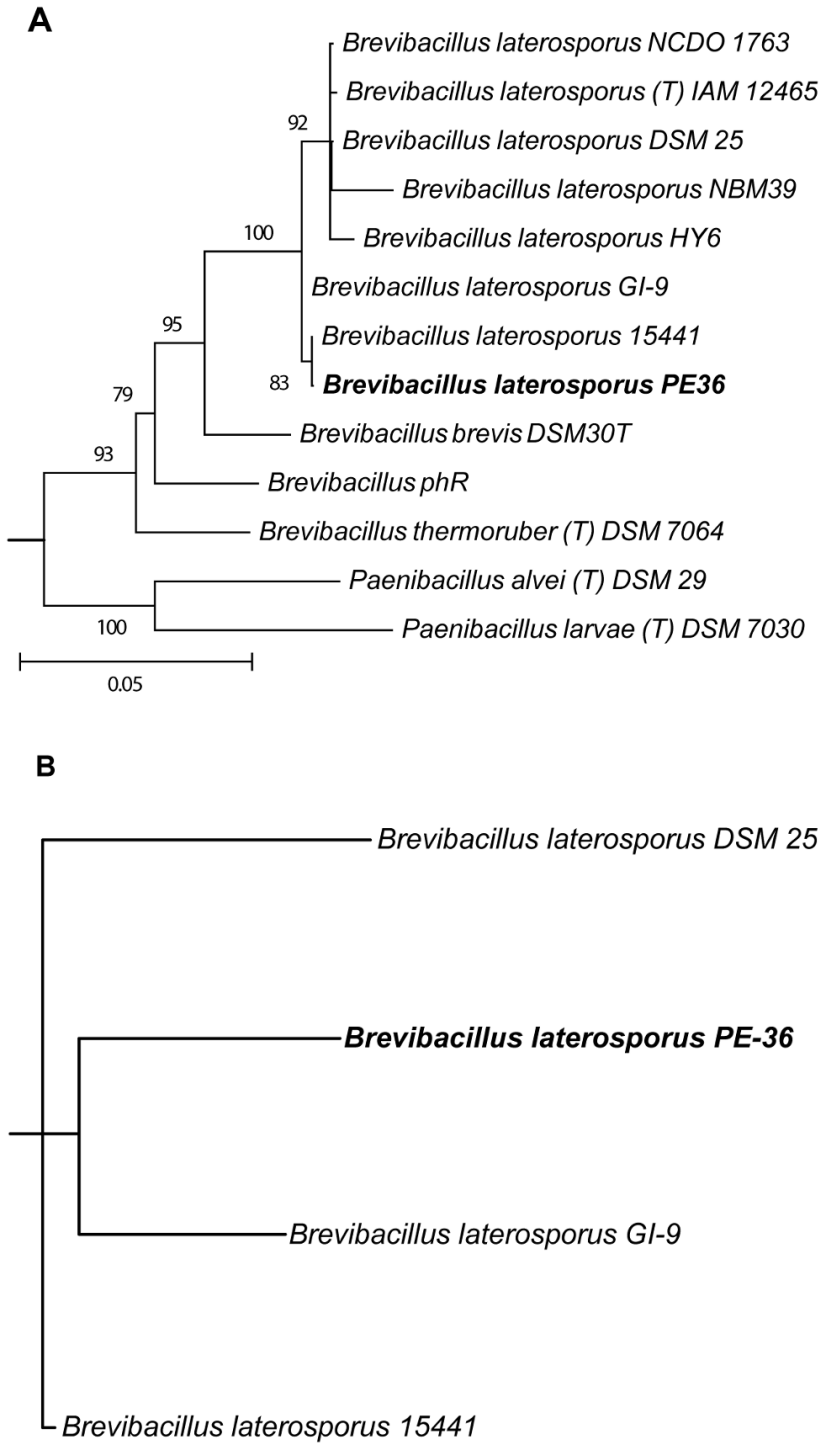
Phylogeny of *B. laterosporous* PE36 and several related *B. laterosporous* isolates. Phylogeny is depicted by (**A**) maximum likelihood of 16S rRNA gene sequences and (**B**) a consensus network based on whole genome comparisons. Bootstrap values >70% are listed for nodes in the maximum likelihood tree with the scale bar representing 0.05 substitutions per position. Branch lengths for the genome consensus network are proportional to the number of likely inversion events of least collinear blocks inferred by Mauve.

Genomic analysis of isolate PE36 yielded a total of 16.75 million reads or 2.49 Gbp with 99.8 percent of reads passing quality filtering (>Q30 across a sliding window of 50 bp). A total of 63 scaffolds with a genome size of 5.14 Mbp were obtained upon assembly. These scaffolds had an N50 of 155.81 Kbp and a maximal scaffold length of 457.91 Kbp producing a near-complete genome assembly (489-fold coverage). After annotation, 4,791 coding sequences were split between 430 subsystems along with 100 RNAs. The complete 16S small subunit rRNA sequence was used to identify isolate PE36 as a member of the family Paenibacillaceae and the genus *Brevibacillus* (Figure 2A). A sequence-based comparison was conducted between the genomes of strain PE36 and the other sequenced *Brevibacillus laterosporus* strains (LMG15441, GI-9, and DSM 25). The genome of *Brevibacillus* phR was included due to its close and unresolved phylogenetic relationship among strains of *B. laterosporus*. This analysis warranted the designation of PE36 as a separate strain of *B. laterosporus* (Figure 2B) and its genome was submitted to the NCBI GenBank (accession number NZ_AXBT00000000). The genome of *B. laterosporus* strain PE36 was evaluated in the secondary metabolite analysis pipeline antiSMASH [18] revealing 32 possible biosynthetic gene clusters. This included 11 nonribosomal peptide synthase (NRPS) clusters, two polyketide synthase (PKS) clusters, and four hybrid pathways (Table 1).

**Table 1:** Secondary metabolite biosynthetic gene clusters identified by antiSMASH.^a^

Cluster #	Type	Contig #	Location	Length (bp)
**1**	Bacteriocin	38	80488 - 94436	13948
**2**	NRPS	53	1 - 40768	40767
**3**	NRPS/T1PKS/terpene	54	1 - 33107	33106
**4**	NRPS	11	26348 - 87628	61280
**5**	NRPS	11	37970 - 121360	83390
**6**	Possible gene cluster	11	125350 - 145149	19799
**7**	Possible gene cluster	27	47359 - 64591	17232
**8**	Possible gene cluster	12	80286 - 110252	29966
**9**	Possible gene cluster	34	48 - 5440	5392
**10**	Siderophore	1	96334 - 110033	13699
**11**	Bacteriocin/T2PKS/NRSP/*trans*-AT PKS	30	1 - 98694	98693
**12**	*Trans*-AT PKS	20	82148 - 145314	63166
**13**	Other	48	1 - 24930	24929
**14**	NRPS	19	1 - 73884	73882
**15**	NRPS/*trans*-AT PKS	19	2773 - 124967	122194
**16**	Possible gene cluster	3	131911 - 144532	12621
**17**	NRPS	21	1 - 39457	39456
**18**	Bacteriocin	32	49076 - 61013	11937
**19**	Possible gene cluster	4	5209 - 19315	14106
**20**	Possible gene cluster	4	248519 - 260263	11744
**21**	Possible gene cluster	10	31262 - 47534	16272
**22**	NRPS	10	154721 - 205832	51111
**23**	NRPS	25	18455 – 82131	63676
**24**	NRPS	7	1 - 36763	36762
**25**	NRPS	7	62273 - 99504	37231
**26**	NRPS/*trans*-AT PKS	8	27546 - 139639	112093
**27**	T3PKS	8	293580 - 334635	41055
**28**	NRPS	2	8150 - 97503	89353
**29**	NRPS	16	32087 - 127019	94932
**30**	Possible gene cluster	14	480 - 9230	8750
**31**	Phosphonate	14	159394 - 200281	40887
**32**	Possible gene cluster	14	229110 - 233512	4402

a Secondary metabolite gene clusters predicted by the antiSMASH platform. Gene clusters with strong homologies to families of known biosynthetic gene cluster types are tentatively assigned (e.g., NRPS, etc.). Gene clusters with biosynthetic characteristics lacking well defined similarities to known types of biosynthetic gene clusters are labeled “Possible gene cluster.” NRPS: nonribosomal peptide synthetase, PKS: polyketide synthase, T1PKS: type I PKS, T2PKS: type II PKS, T3PKS: type III PKS.

**Figure 3:**
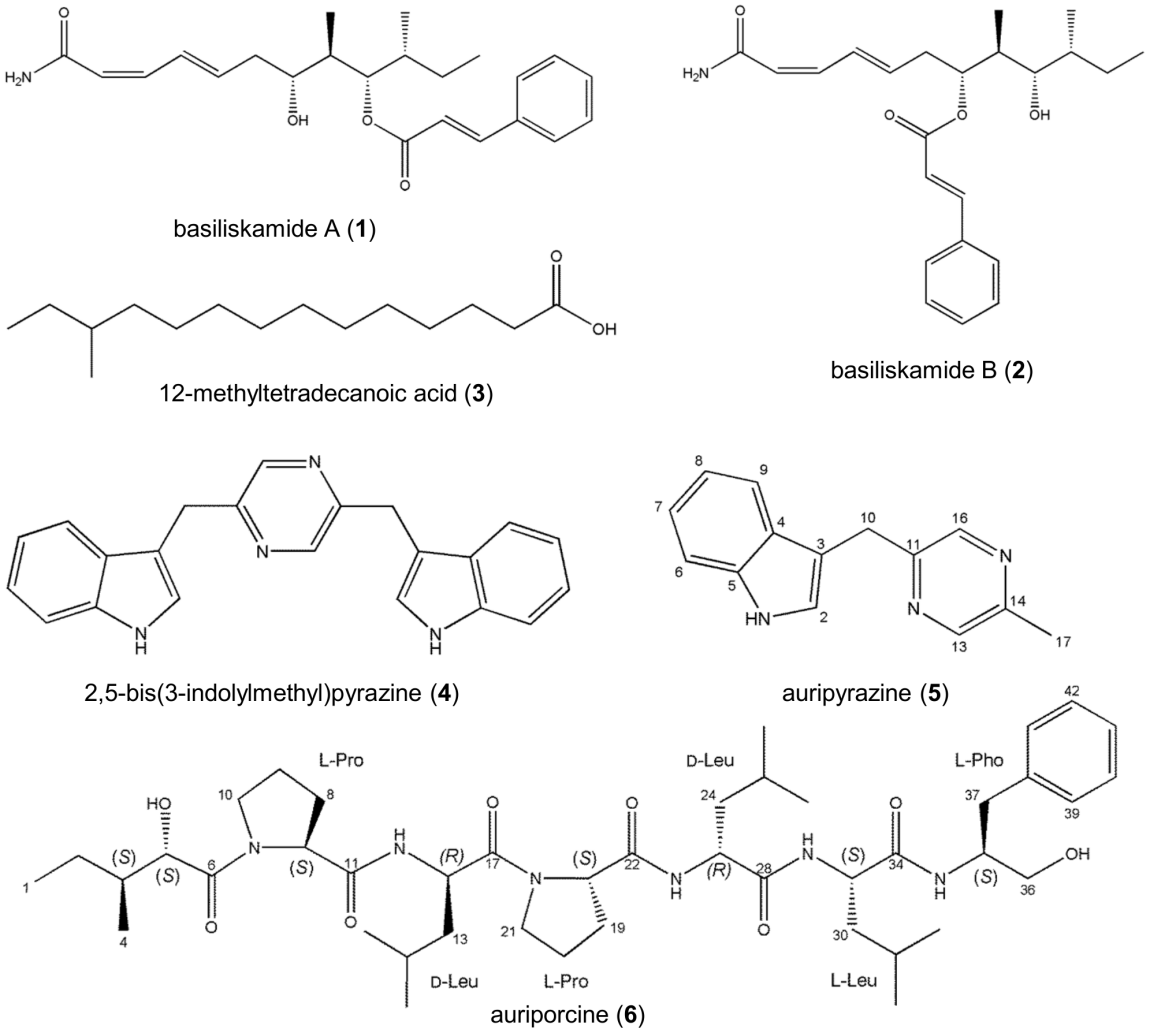
Structures of secondary metabolites isolated from *B. latersporus* PE36.

A scale-up culture of *B. laterosporus* PE36 was prepared for the purification and structure characterization of its natural products. Silica flash chromatography, C_18_ vacuum liquid chromatography (VLC), and preparative and semi-preparative C_18_ reversed-phase high performance liquid chromatography (RP-HPLC) were used to purify the metabolites (Figure 3) responsible for the extract's biological activities. This yielded two bioactive (antibacterial) fractions. The first active fraction was determined to contain basiliskamides A (**1**) and B (**2**) [19], the structures of which were confirmed by high-resolution electrospray ionization mass spectrometry (HRESI-MS) and ultraviolet (UV) spectroscopy, as well as one-dimensional proton nuclear magnetic resonance spectroscopy (^1^H NMR) and two-dimensional (2D) heteronuclear single quantum coherence spectroscopy (^1^H-^13^C HSQC) data. Compounds **1** and **2** exhibit potent antifungal activities and modest antibacterial properties [20]. These compounds were previously reported to have been produced by a strain of *Brevibacillus* associated with a marine tubeworm collected in Papua New Guinea [19]. The active component from the second active fraction was identified by gas-chromatography-mass-spectrometry (GC-MS) and ^1^H NMR as 12-methyltetradecanoic acid (**3**) [21].

To further probe the chemical diversity of the *B. laterosporus* PE36 metabolome, two additional fractions that lacked antibacterial activities were selected for chemical analysis. Liquid-chromatography-mass-spectrometry (LC-MS) profiling of the first inactive fraction revealed the presence of two compounds possessing similar UV absorbance spectra (λ_max_ ∼220). One of the compounds was identified as 2,5-bis(3-indolylmethyl)pyrazine (**4**), whereas the second compound (**5**) is reported here for the first time as a natural product. High resolution ESI-MS analysis of compound **5** yielded a molecular ion at *m/z* 222.1039 [M-H]^-^, which substantiated a molecular formula of C_14_H_13_N_3_. The ^1^H and ^13^C NMR data for compound **5** were very similar to those obtained for compound **4** (Table 2). Analysis of the ^1^H NMR data revealed a broad exchangeable singlet at δ_H_ 10.9, two downfield singlets at δ_H_ 8.32 and 8.36, five aromatic protons (δ_H_ 7.22 – 6.94), a singlet integrating for two hydrogens at δ_H_ 4.17, and a methyl singlet at δ_H_ 2.45. These features could be accounted for if one of the two indoles attached to the pyrazine in compound **4** was replaced by a methyl group in compound **5**. The proposed structural change was subsequently confirmed by 2D NMR to establish the structure of auripyrazine (**5**).

**Table 2:** ^1^H (500 MHz) and ^13^C (125 MHz) NMR data (DMSO-*d*_6_, 25°C) for auripyrazine (5).

Position	^13^C, type^a^	^1^H (*J* in Hz)
**2**	123.7, CH	7.22, m
**3**	111.1, C	-
**4**	125.7, C	-
**5**	136.1, C	-
**6**	111.2, CH	7.33, d (8.2)
**7**	119.0, CH	6.94, m
**8**	119.0, CH	7.48, m
**9**	121.5, CH	7.06, m
**10**	31.3, CH_2_	4.17, s
**11**	155.4, C	-
**13**	142.3, CH	8.32, s
**14**	152.3, C	-
**16**	141.6, CH	8.35, s
**17**	21.8, CH_3_	2.45, s
**1-N** ***H***	-	10.92, brs

a 13 C data were obtained *via* inverse detection ^1^H-^13^C HSQC and ^1^H-^13^C gHMBC experiments.

HRESI-MS analysis of compound **6** provided a molecular ion with *m/z* 799.5330 [M+H]^+^, which indicated that the compound had a molecular formula of C_43_H_70_N_6_O_8_. An initial inspection of the ^1^H NMR data revealed that compound **6** was likely peptidic in nature with signals characteristic of amide doublet protons, as well as amino-acid α-hydrogen spins (Table 3). Further inspection of the 1D and 2D NMR data revealed the occurrence of many more signals than could be accounted for by the proposed molecular formula; however, additional attempts at purification of the compound by analytical RP-HPLC were unsuccessful. We also observed by ^1^H NMR that the relative distribution of these spins was not appreciably altered by switching to different solvents (i.e., DMSO-*d*_6_, acetone- *d*_6_, CDCl_3_, MeOH-*d*_4_, and pyridine-*d*_5_) or upon changes to the NMR probe temperature. Therefore, we conjectured that compound **6** might exist in two dominant conformational states (Table 3) that were relatively insensitive to the influence of the surrounding solvent.

**Table 3:** ^1^H (500 MHz) and ^13^C (125 MHz) NMR data (DMSO-*d*_6_, 25°C) for the two predominant solution conformers of auriprocine (6).

Residue	Position	Conformer 1		Conformer 2	
		^13^C^a^	^1^H	^13^C^a^	^1^H
**SCF^b^**	1	11.1, CH_3_	0.82	11.1, CH_3_	0.82
	2	22.8, CH_2_	1.52, m; 1.06, m	22.8, CH_2_	1.51, m; 1.08, m
	3	37.1, CH	1.66, m	37.1, CH	1.67, m
	4	15.0, CH_3_	0.86, m	15.0, CH_3_	0.86, m
	5	72.5, CH	3.91, m	72.5, CH	3.94, m
	6	172.6, C	-	172.6, C	-
**L-Pro**	7	59.1, CH	4.84, m	59.1, CH	4.82, m
	8	31.5, CH_2_	2.09, m	31.5, CH_2_	2.09, m
	9	28.5, CH_2_	1.75, m	28.5, CH_2_	1.75, m
	10	46.2, CH_2_	3.41, m	46.2, CH_2_	3.41, m
	11	171.9, C	-	171.9, C	-
**D-Leu**	NH	-		-	
	12	51.3, CH	4.35, m	51.3, CH	4.36, m
	13	39.9, CH_2_	1.45, m	39.6, CH_2_	1.25, m
	14	23.9, CH	1.53, m	23.7, CH	1.47, m
	15	20.8-23.0, CH_3_	0.86, m	20.8-23.0, CH_3_	0.76, m
	16	20.8-23.0, CH_3_	0.76, m	20.5, CH_3_	0.66, m
	17	170.9-172.2	-	-	
**L-Pro**	18	60.0, CH	4.25, m	59.7, CH	4.34, m
	19	28.9, CH_2_	2.05, m	28.9, CH	2.05, m
	20	24.1, CH_2_	1.78, m; 1.91, m	24.1, CH_2_	1.78, m; 1.91, m
	21	46.4, CH_2_	3.67, m; 3.48, m	46.4, CH	3.65, m; 3.50, m
	22	171.6	-	171.6	-
**D-Leu**	NH	-	7.83, m	-	7.84, m
	23	48.6, CH	4.53, m	51.5, CH	4.17, m
	24	39.6, CH_2_	1.45, m	39.6, CH_2_	1.48, m
	25	23.6, CH	1.56, m	23.6, CH	1.51, m
	26	20.8-23.0, CH_3_	0.87, m	20.8-23.0, CH_3_	0.82, m
	27	20.8-23.0, CH_3_	0.87, m	20.8-23.0, CH_3_	0.87, m
	28	170.3, C	-	170.9-172.2, C	-
**L-Leu**	NH	-	8.00, d (8.3)	-	8.15, m
	29	51.6, CH	4.15, m	50.6, CH	4.21, m
	30	40.9, CH_2_	1.41, m	40.0, CH	1.38 m
	31	23.7, CH	1.50, m	23.7, CH	1.45, m
	32	20.8-23.0, CH_3_	0.84, m	20.8-23.0, CH_3_	0.86, m
	33	20.8-23.0, CH_3_	0.78, m	20.8-23.0, CH_3_	0.79, m
	34	170.9-172.2, C	-	170.9-172.2, C	-
**L-Pho**	NH	-	7.57, d (8.8)	-	7.67, d (8.3)
	35	52.6, CH	3.86, m	52.6, CH	3.86, m
	36	62.4, CH	3.30, m; 3.25,m	62.36, CH	3.29, m; 3.21, m
	37	36.5, CH	2.86, m; 2.67, m	36.5, CH	2.85, m; 2.65, m
	38	139.0, C	-	139.0, C	-
	39	128.6, CH	7.21, m	128.6, CH	7.21, m
	40	127.7, CH	7.25, m	127.7, CH	7.25, m
	41	125.5, CH	7.17, m	125.5, CH	7.14, m
	42	127.7, CH	7.25, m	127.7, CH	7.25, m
	43	128.6, CH	7.21, m	128.6, CH	7.21, m

a 13 C data were obtained *via* inverse detection by ^1^H-^13^C HSQC and ^1^H-^13^C gHMBC experiments. ^b^SCF: six carbon fragment.

Analysis of the data from 2D proton total correlation spectroscopy (^1^H-^1^H TOCSY), 2D gradient heteronuclear multiple bond correlation spectroscopy (^1^H-^13^C gHMBC), and 2D nuclear Overhauser effect spectroscopy (^1^H-^1^H NOESY) experiments provided evidence for several discrete spin systems attributable to two proline and three leucine residues that were linked to form a peptidic Pro-Leu-Pro-Leu-Leu fragment. Another spin set was probed in greater detail with ^1^H-^13^C gHMBC leading to the generation of a six carbon fragment in which two of the carbon atoms were attached to oxygen atoms (δ_C_ 172.0 and 72.5, amide carbonyl and a hydroxyl-group-bearing methine, respectively) (Figure S1). Examination of the ^1^H-^13^C gHMBC data helped link this fragment to the peptide portion of the new metabolite (Figure S1). The placement of this six carbon fragment at the *N*-terminus of the peptide was further supported by a one-bond proton-nitrogen heteronuclear correlation (^1^H-^15^N HSQC) experiment from which we found no evidence for primary amide hydrogens. The remaining carbon atoms in compound **6** were determined to comprise a phenylalaninol residue (Pho) at the *C*-terminus of the peptide (Figure S1). The reduction of the L-phenylalanine carboxyl group to a primary alcohol was confirmed by the presence of a carbon chemical shift at δ_C_ 62.4 for the atom bearing the primary alcohol. A series of MS*^n^* experiments (Table 4) were performed to provide additional substantiation for the proposed planar structure of compound **6**. A majority of the fragment ions exhibited a loss of water and/or putative intramolecular cyclization as reported for similar structures [22]. Several of the fragments were generated through cleavage of the amide bonds, thus confirming the proposed sequence of amino acid residues in compound **6**.

**Table 4:** MS*^n^* fragments used to support the planar structure of auriprocine (6).^a^

Fragment	Parent ions, *m/z*	Fragment *m/z*
**[SCF-Pro-Leu-Pro-Leu]^+^**	799, 781	535
**[SCF-Pro-Leu-Pro]^+^**	799, 781, 535	422
**[Pro-Leu-Leu-Pho]^+^ + 2H**	799, 781	457
**[Leu-Leu-Pho]^+^ + 2H**	799, 781, 457	360
**[Leu-Pho]^+^ + 2H**	799, 781, 457	247

a The *m/z* of the parent ion of each fragment is shown. SCF: *N*-terminal six carbon fragment, Pho: phenylalaniol.

**Figure 4:**
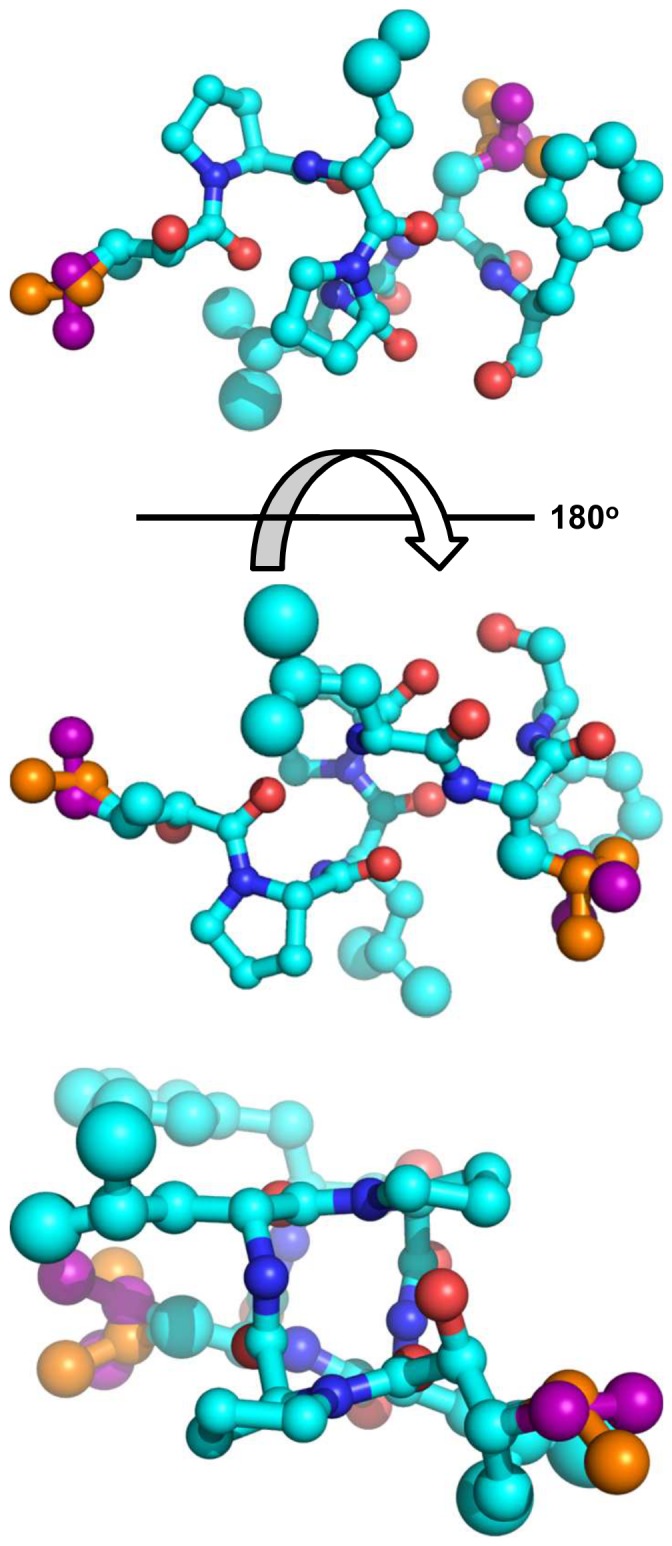
Pymol representation of auriprocine (6). Images generated from X-ray data for **6**. The top two images depict the two opposing faces of the structure, rotated 180°. The bottom image is the helical wheel representation, viewed from the *N*-terminus along the helix axis. An animated image of **6** is provided in Movie S1.

The relative configuration of compound **6** was determined by X-ray diffraction experiment on a single crystal prepared in a vapor diffusion chamber (acetone and ether) (Table S1). Compound **6** exhibited a helix-like conformation in the crystalline state with the leucine residue side chains projecting outward from the compound in a relatively disordered state (Figure 4). We observed a 3S*,5S*,7S*,12R*,18S*,23R*,29S*,35S* relative configuration for **6**, which indicated that some of the incorporated amino acid residues possessed a D-configuration. Marfey's analysis [23] was subsequently carried out demonstrating that both of the proline residues were L-configured (Figure S2). In contrast, we detected a ∼2∶1 mixture of D- and L-leucines, respectively, using the Marfey's derivatization method. This was in agreement with the proposed 12R*, 23R*, 29S* assignments for the three leucine residues as determined by X-ray analysis (Figure S2). The Marfey's analysis also provided definitive evidence for the presence of L-phenylalaninol (Figure S2). Thus, the absolute configuration of compound **6** was determined to be 3*S*,5*S*,7*S*,12*R*,18*S*,23*R*,29*S*,35*S*.

**Figure 5:**
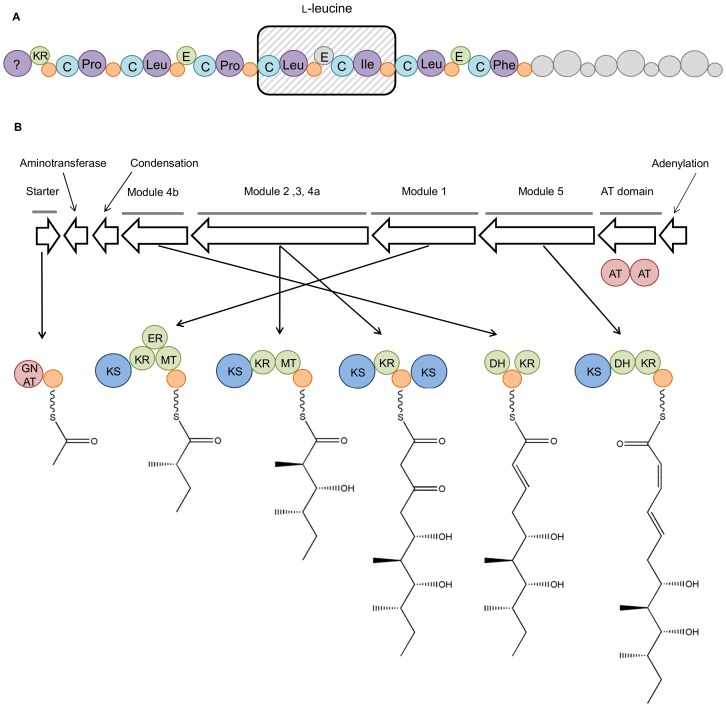
Interpretation of antiSMASH results and proposed biosynthetic origins of basiliskamides A (1) and B (2) and auriporcine (6). Panel A illustrates the biosynthetic gene cluster hypothesized to be responsible for the production of 6. Adenylation domains are labeled with the amino acids they are predicted to contribute. The two domains potentially involved in loading of the L-leucine residues are contained in the shaded box. The additional domains shaded grey are believed not to be involved in the production of 6 or were incorporated, but later removed from the metabolite. Panel B shows the organization of the putative basiliskamide gene cluster, as well as a proposed biosynthetic scheme for the production of the polyketide portions of 1 and 2. The fused, tandem AT domains are separate from the other modules. The dehydrating bimodule is shown split, as the gene that encodes the two KS domains are separated from the dehydrogenase and ketoreductase. The genes encoding the predicted aminotransferase, condensation, and adenylation domains (hypothesized to transform the amide and add the cinnamic acid moieties to the polyketide chain) are labeled. The putative functions of the genes are indicated as A (adenylation), C (condensation), KR (ketoreductase), E (epimerization), GNAT (GCN5-related acetyltransferase), KS (ketosynthase), ER (enolreductase), MT (methyltransferase), DH (dehydrogenase), and AT (acetyltransferase).

The genome of *B. laterosporus* PE36 was reexamined to provide a link between the secondary metabolites made by this isolate and their respective biosynthetic genes. Of the 11 NRPS gene clusters identified in PE36 (Table 1), cluster 23 (Figure 5A) exhibited several distinctive features making it the likely source of compound **6**. Namely, cluster 23 contains an initial PKS-like initiation module that is postulated to be responsible for loading the non-amino acid starter unit at the compound's *N*-terminus. This is followed by NRPS modules containing adenylation domains that are predicted to sequentially incorporate proline → leucine → proline → leucine → isoleucine → leucine → tyrosine → leucine → isoleucine → threonine. The observed order of amino acids in compound **6**, L-proline → D-leucine → L-proline → D-leucine → L-leucine → L-phenylalaniol, fits reasonably well with this prediction. Upon closer inspection, epimerization domains were identified as being associated with each of the predicted leucine incorporation steps. This explains the D-configurations of the first two leucines. In contrast, the L-configuration of the third leucine is hypothesized to have resulted from an inactive epimerization domain or the leucine incorporation module is skipped and the downstream isoleucine incorporation module that lacks an epimerization domain installs the final leucine. The termination of the NRPS chain at the tyrosine/phenylalanine residue could occur via several scenarios including 1) phenylalanine incorporation followed by termination and post-production reductive tailoring; 2) tyrosine incorporation followed by termination, dehydroxylation, and reduction; or 3) full incorporation of all the predicted amino acids followed proteolysis of a tyrosine/phenylalanine → leucine bond and reduction. Based on the order of amino acid residues predicted by antiSMASH analysis and presence of appropriate modifying features (i.e., epimerization domains), putative biosynthetic gene cluster 23 is most likely responsible for the production of **6**; however, further experiments will be needed to confirm this hypothesis.

The antiSMASH data were also probed to determine the probable gene cluster responsible for the production of the basiliskamides (**1** and **2**). Focusing on the only two gene clusters predicted to be predominatly composed of PKS-related domains (Table 1), we propose that biosynthetic cluster 12 is the most likely candidate for the production of compounds **1** and **2**. Cluster 12 is predicted to be a *trans*-AT PKS, which also contains an NRPS-like domain. The *trans*-AT PKSs are architecturally and biosynthetically unique in a number of key ways [24]. Most notably, none of the individual modules in a *trans*-AT PKS contain a dedicated AT (acetyltransferase) domain. Instead the modules receive carbon building blocks from a shared AT, which in this case is a fused tandem AT domain [25]. Figure 5B illustrates the genomic organization of the proposed biosynthetic gene cluster and a plausible biosynthetic pathway responsible for the generation of compounds **1** and **2**. The starter unit is loaded by a predicted acetyl-loading AT of the GCN5-related superfamily (GNAT) [26]. Each ketosynthase domain (KS) is expected to use malonyl-CoA to build the polyketide chain. Ketoreductases (KR), enolreductases (ER), and methyl transferases (MT) serve to further derivatize the polyketide. The fourth module is split with a domain configuration of KS – KR – ACP – KS – DH – ACP – KR, which is indicative of type B dehydrating bimodules [25]. These types of modules are capable of producing double bonds with either the *E* or *Z* configurations [25].

The non-polyketide portions of compounds **1** and **2** are likely introduced from three additional genes co-located within the gene cluster. One of these genes was identified as a putative aminotransferase that could be responsible for converting a carboxylate into an amide [7]. The cinnamic acid portion of the molecule could be installed by an NRPS-like adenylation domain. Adenylation domains have previously been shown to incorporate non-amino acid moieties into compounds *via* ester linkages [6], [27]. This assessment of gene cluster 12 represents a theoretical basis for understanding the biosynthetic origins of **1** and **2**. Further experiments including feeding studies, gene knockouts, and other molecular manipulation approaches will be required to confirm these hypothetical steps leading to the production of **1** and **2**.

The results of these combined chemical and genomic studies suggest that bacteria associated with wild mammals are a potential source of unique and bioactive secondary metabolites. Despite the promising opportunities revealed through this work, we can only speculate about the nature of the biological relationship between *B. laterosporus* PE36 and feral hogs (e.g., is the bacterium common to the host or an example of a transient association). Our group has initiated studies to investigate this and related questions about the bacteria recovered from the microbiomes of wild mammals. Nevertheless, our past and current screening efforts involving other wild mammals and their associated bacteria suggest that these microbes have tremendous potential for accessing new dimensions of microbial biodiversity and chemodiversity.

## Materials and Methods

### General Experimental

UV data were collected on a Hewlett Packard 8452A diode array spectrophotometer. Optical rotation data were determined on a Rudolph Research Analytical Autopol III automatic polarimeter. UV–CD spectra were measured on an AVIV circular dichroism spectrometer model 202-01. NMR data were obtained on a Varian VNMR spectrometer (500 MHz). Accurate mass data were collected on an Agilent 6538 HRESI QTOF MS coupled with an Agilent 1290 HPLC. LC-MS analyses were performed on a Shimadzu LC-MS 2020 system (ESI quadrupole) coupled to a photodiode array detector. The samples were separated using a Phenomenex Kintex column (2.6 µm C_18_ column, 100 Å, 75×3.0 mm). The HPLC system utilized SCL-10A VP pumps and system controller with a Gemini 5 µm C_18_ column (110 Å, 250×21.2 mm, flow rates of 1 to 10 mL/min). X-ray data were collected using a diffractometer with a Bruker APEX ccd area detector and graphite-monochromated Mo Kα radiation (λ = 0.71073 Å). All solvents were HPLC grade or better.

### Collection and Isolation of Bacteria

The mouth, nose, and ears of a feral hog, shot by a hunter (in compliance with state ordinances) in southwestern Oklahoma, were swabbed with sterile cotton swabs. Since our opportunistic sampling strategy does not involve the handling of live animals, only sampling carcasses, we have been assigned an internal case-tracking number (R11-021) by the University of Oklahoma IACUC. A State-of-Oklahoma-issued Scientific Collector Permit (permit #5250) was obtained for the purpose of sampling carcasses. Permission was granted to sample the head of the hog carcass; the research team did not take ownership of the carcass at any time. The tip of each swab was removed and placed into a sterile 15 mL Falcon tube containing a 0.9% NaCl (w/v) solution. The tubes were vortexed and diluted 1∶10 with 0.9% NaCl. Aliquots of 50 µL were spread on half-strength tryptic soy agar (TSA) plates. The plates were incubated at 37°C under a 5% CO_2_ atmosphere for two weeks. Colonies were selected based on morphological uniqueness and re-streaked on half strength TSA plates until pure, morphologically homogenous cultures were obtained.

### Overlay Assay

Bacterial isolates were arrayed onto half strength TSA plates and incubated for up to one week. A layer of half strength TSA seeded with methicillin-resistant *S. aureus* was applied to the surface of each plate. The plates were incubated at 37°C under a 5% CO_2_ atmosphere for five days and periodically examined for zones of *S. aureus* inhibition.

### DNA Extraction and Sequencing

Genomic DNA was extracted using the PowerBiofilm DNA Isolation kit (MoBio Laboratories, Carlsbad, CA) by spinning down 2.0 mL of turbid culture and following the manufacturer instructions. The purity of gDNA was confirmed by spectrophotometery (Implen) and submitted to the Oklahoma Medical Research Foundation genomics core facility for sequencing on an Illumina MiSeq using TruSeq LT 2×150 bp chemistry (Illumina, San Diego, CA). Reads were assembled using the CLC Genomics Workbench suite *de novo* assembly algorithm (CLC Bio, Cambridge, MA). Contigs smaller than 800 bp were discarded. After assembly, scaffolds were initially submitted to the RAST server for total-genome annotation [28]. For final annotation, the scaffolds were submitted to the NCBI PGAP server. RAST annotated scaffolds were also uploaded to antiSMASH [18] to identify putative biosynthetic gene clusters associated with the production of secondary metabolites. Pathways identified by antiSMASH were amended to the PGAP annotation after submission to GenBank. Annotated gDNA was deposited with GenBank under the accession number NZ_AXBT00000000.

### Culture Conditions and Extraction

Starter cultures of the bacterium were prepared in tryptic soy broth (TSB) and shaken on a rotary shaker at 130 rpm at room temperature for 12 h. For scale-up preparation, 1 L Erlenmeyer flasks containing 300 mL of sterile TSB were inoculated with 1 mL of starter culture. The flasks were shaken at 130 rpm at room temperature for 1 week and the cultures were pooled prior to partitioning. The pooled culture broth with cells was partitioned three times against EtOAc (1∶1 vol/vol) and the solvent removed from the organic layer under reduced pressure on a rotary evaporator.

### Compound Isolation

The crude extract (∼4 g from 30 L culture) was absorbed onto silica gel and subjected to flash chromatography fractionation using a hexane-CH_2_Cl_2_-MeOH gradient on an Isolera System (Biotage, Charlotte, NC). The purification of each compound was achieved as follows:

#### 12-methyltetradecanoic acid (3)

Silica flash column chromatography was performed (hexane-DCM-MeOH) and the fraction that eluted with 100% DCM was further separated using C_18_ RP-HPLC. A MeOH-H_2_O gradient (20% MeOH for 5 minutes, 20–100% MeOH gradient over 50 minutes, 100% MeOH for 10 minutes at a 10 mL/min flow rate) resulted in a single active fraction. This fraction was purified by semi-preparative RP-HPLC (60% acetonitrile for 10 minutes, 60–100% acetonitrile gradient over 45 minutes, 100% acetonitrile for 10 minutes at 2 mL/min) to provide **3** (10 mg). The structure of the metabolite was determined by ^1^H NMR and GC-MS analysis.

#### Basiliskamides A (1) and B (2)

C_18_ VLC (step-gradient of 25∶75, 50∶50, 75∶25, and 100∶0 MeOH-H_2_O) was performed and the fraction that eluted with 100% MeOH was further separated by C_18_ RP-HPLC. A MeOH-H_2_O gradient (20% MeOH for 5 minutes, 20–100% MeOH gradient over 50 minutes, 100% MeOH for 10 minutes at a 10 mL/min flow rate) resulted in a single active fraction. This fraction was further processed by semi-preparative C_18_ RP-HPLC (isocratic, 80% MeOH) to yield **1** and **2** (2 and 6 mg, respectively). The compounds were identified based on comparisons of their experimental and published HR-ESIMS, UV, and NMR data [19].

#### 2,5-bis(3-indolylmethyl)pyrazine (4)

An HPLC fraction that eluted after **1** and **2** was collected and separated by C_18_ RP-HPLC (60% MeOH for 5 minutes, 60–100% MeOH over 50 minutes, 100% MeOH for 10 minutes at a 10 mL/min flow rate) to provide **4** (7 mg). The structure of **4** was determined by comparing its HR-ESIMS and ^1^H NMR data to published values [29].

#### Auripyrazine (5)

A fraction obtained prior to the elution of **1** and **2** was collected and fractionated by preparative C_18_ RP-HPLC (20% MeOH for 5 minutes, 20–100% MeOH gradient over 50 minutes, 100% MeOH for 10 minutes at a 10 mL/min flow rate). Purification by semi-preparative C_18_ RP-HPLC (isocratic, 40% acetonitrile with 0.1% formic acid at a 2 mL/min flow rate) provided **5** (5 mg).

#### Auriporcine (6)

A late eluting fraction from the same C_18_ RP-HPLC gradient that yielded **1** and **2** was pursued for further analysis. Semi-preparative C_18_ RP-HPLC with acetonitrile and H_2_O treated with 0.1% trifluoroacetic acid (10% acetonitrile for 5 minutes, 10–100% acetonitrile gradient over 55 minutes at a 2 mL/min flow rate) resulted in the purification of **6** (2 mg).

### Marfey's Analysis

Marfey's analysis [23] was performed to determine the absolute configuration of each amino acid contained in **6**. Briefly, approximately 1 mg of **6** was dissolved in 6 M HCl and heated at 100°C for approximately 18 h. After heating, the sample was dried under reduced pressure, redissolved in 50 µL of water and transferred to a 1.5 mL centrifuge tube. Aliquots consisting of 50 µL of 50 mM of the D and L enantiomers of each amino acid were independently treated in 1.5 mL centrifuge tubes. To each standard and sample, 100 µL of 1% FDAA and 20 µL of 1 M NaHCO_3_ were added. Tubes were capped and heated in a 40°C water bath with periodic mixing for 1 h. After cooling to room temperature, 10 µL of 2 M HCl was added to each tube. The mixtures were dried under reduced pressure and redissolved in 200 µL of 9∶1 MeOH-H_2_O. Samples were diluted further to one tenth of their original concentration with 9∶1 MeOH-H_2_O prior to LC-MS analysis.

### X-ray Analysis

A colorless prism-shaped crystal of **6** with dimensions 0.44×0.16×0.12 mm was selected for structure analysis. The sample was cooled to 100 K. Cell parameters were determined from a non-linear least squares fit of 6,210 peaks in the range 2.41<θ<26.04°. A total of 40,661 data points were measured in the range 1.554<θ<28.344° using φ and ω oscillation frames. The data were corrected for absorption by the empirical method giving minimum and maximum transmission factors of 0.964 and 0.990. The data were merged to form a set of 10,800 independent data with R(int)  = 0.0586 and a coverage of 99.9%. The orthorhombic space group P2_1_2_1_2_1_ was determined by systematic absences and statistical tests and verified by subsequent refinement. The structure was solved by direct methods and refined by full-matrix least-squares methods on F^2^. The positions of hydrogens bonded to carbons were initially determined by geometry and were refined using a riding model. Hydrogens bonded to nitrogens and oxygens were located on a difference map, and their positions were refined independently. Non-hydrogen atoms were refined with anisotropic displacement parameters. Hydrogen atom displacement parameters were set to 1.2 (1.5 for methyl) times the isotropic equivalent displacement parameters of the bonded atoms. A total of 573 parameters were refined against 118 restraints and 10,800 data to give wR(*F* ^2^)  = 0.1925 and S = 1.011 for weights of w = 1/[σ2 (*F* ^2^) + (0.0900 P)^2^ + 5.0000 P], where P = [*F*_o_^2^ + 2*F*_c_^2^]/3. The final R(*F*) was 0.0707 for the 9,029 observed, [*F*>4σ (*F*)], data. The largest shift/s.u. was 0.012 in the final refinement cycle. The final difference map had maxima and minima of 0.315 and -0.374 e/Å^3^, respectively. The absolute structure could not be determined by refinement of the Flack parameter. Further documentation of these data can be obtained in CIF File S1.

### Compound Characterization

**Auripyrazine** (**5**): yellow solid; UV (MeOH) λ_max_ 222 (log ε 3.49); ^1^H and ^13^C NMR data refer to Table 2; HRESI-MS [M-H]^-^ *m/z* 222.1039 (calculated for C_14_H_12_N_3_, 222.1031). See Figures S3–S7 for 1D and 2D NMR, HRESI-MS, and UV data.

**Auriporcine** (**6**): white solid; UV (MeOH) λ_max_ 206 (log ε 4.19), [α]_D_ (*c* 0.065) -27.7; ^1^H and ^13^C NMR data refer to Table 3; HRESI-MS [M+H]^+^ *m/z* 799.5330 (calculated for C_43_H_71_N_6_O_8_, 799.5333). See Figures S8–S18, for 1D and 2D NMR data, thermal ellipsoid plot, HRESI-MS, UV, and CD data. See Movie S1 for an animated PyMol representation of **6**.

## Supporting Information

Figure S1 **2D NMR correlations used to determine the planar structure of auriprocine, 6.** Important 2D NMR correlations used in the structure elucidation of **6** are shown: ^1^H-^1^H TOCSY (shown as rust colored bonds), ^1^H-^1^H NOESY (illustrated as orange double-headed arrows), and ^1^H-^13^C *g*HMBC (shown as blue single-headed arrows).
(TIF)

Figure S2 **Overlaid selective ion trace chromatograms showing the C_18_ LC-MS results of the Marfey's analysis.** Single ion trace detection of the derivatized amino acids: derivatized proline (blue), leucine (red), and phenylalaninol (green).
(TIF)

Figure S3 **^1^H (DMSO-** ***d*** **_6_, 25°C) spectrum of compound 5.** Signals resulting from impurities are marked with red dots.
(TIF)

Figure S4 **^1^H-^13^C HSQC (DMSO-** ***d*** **_6_, 25°C) spectrum of compound 5.** CH groups are shown in blue, CH_2_ groups are in red.
(TIF)

Figure S5 **^1^H-^13^C gHMBC (DMSO-** ***d*** **_6_, 25°C) spectrum of compound 5.**
(TIF)

Figure S6 **HRESI-MS (negative mode) data for compound 5.**
(TIF)

Figure S7 **UV data for compound 5.**
(TIF)

Figure S8 **^1^H NMR (500 MHz, DMSO-** ***d*** **_6_, 25°C) spectrum of compound 6.**
(TIF)

Figure S9 **^1^H-^13^C HSQC (DMSO-** ***d*** **_6_, 25°C) spectrum of compound 6.** CH groups are shown in blue, CH_2_ groups are in red.
(TIF)

Figure S10 **^1^H-^13^C gHMBC (DMSO-** ***d*** **_6_, 25°C) spectrum of compound 6.**
(TIF)

Figure S11 **^1^H-^13^C TOCSY (DMSO-** ***d*** **_6_, 25°C) spectrum of compound 6.**
(TIF)

Figure S12 **^1^H-^1^H gCOSY (DMSO-** ***d*** **_6_, 25°C) spectrum of compound 6.**
(TIF)

Figure S13 **^1^H-^1^H NOESY (DMSO-** ***d*** **_6_, 25°C) spectrum of compound 6.**
(TIF)

Figure S14 **Overlaid ^1^H-^14^N HSCQ (DMSO-** ***d*** **_6_, 25°C) spectra of compound 6.** Overlay of two NMR experiments. Data from the first experiment, optimized to show NH signals only, are illustrated in red. Data from the second experiment, optimized to show both NH and NH_2_, are illustrated in blue. Data from both experiments show complete overlap indicating there are no secondary amines.
(TIF)

Figure S15 **Thermal ellipsoid plot of compound 6.**
(TIF)

Figure S16 **HRESI-MS (positive mode) of compound 6.**
(TIF)

Figure S17 **UV data for compound 6.**
(TIF)

Figure S18 **CD data for compound 6.**
(TIF)

Table S1 **Crystal data and structure refinement data for compound 6.**
(DOCX)

CIF File S1 **X-ray coordinate data for compound 6.**
(CIF)

Movie S1 **Rotating PyMOL representation of compound 6.**
(MP4)
