# Supplementary figures and images for: Genomic and Metabolomic Insights into the Natural Product Biosynthetic Diversity of a Feral-Hog-Associated Brevibacillus laterosporus Strain
Source: PLoS One. 2014 Mar 3;9(3):e90124. doi: 10.1371/journal.pone.0090124 (PMC3940840; doi:10.1371/journal.pone.0090124)

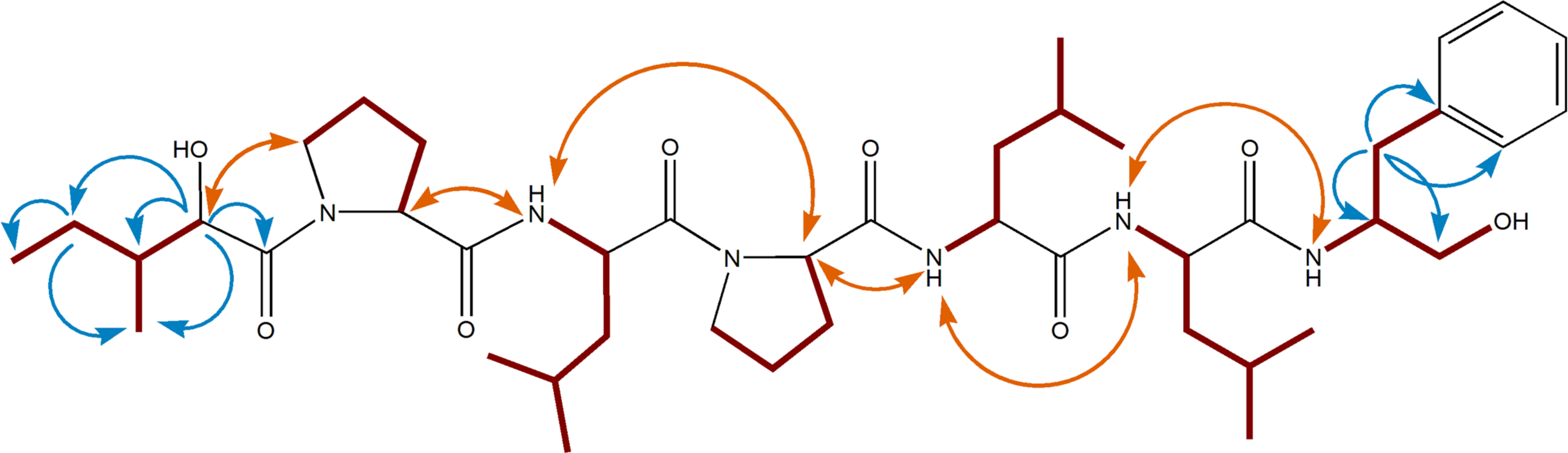

Supplement: Figure S1 — 2D NMR correlations used to determine the planar structure of auriprocine, 6. Important 2D NMR correlations used in the structure elucidation of 6 are shown: 1H-1H TOCSY (shown as rust colored bonds), 1H-1H NOESY (illustrated as orange double-headed arrows), and 1H-13C gHMBC (shown as blue single-headed arrows). (TIF) [file pone.0090124.s001.tif]

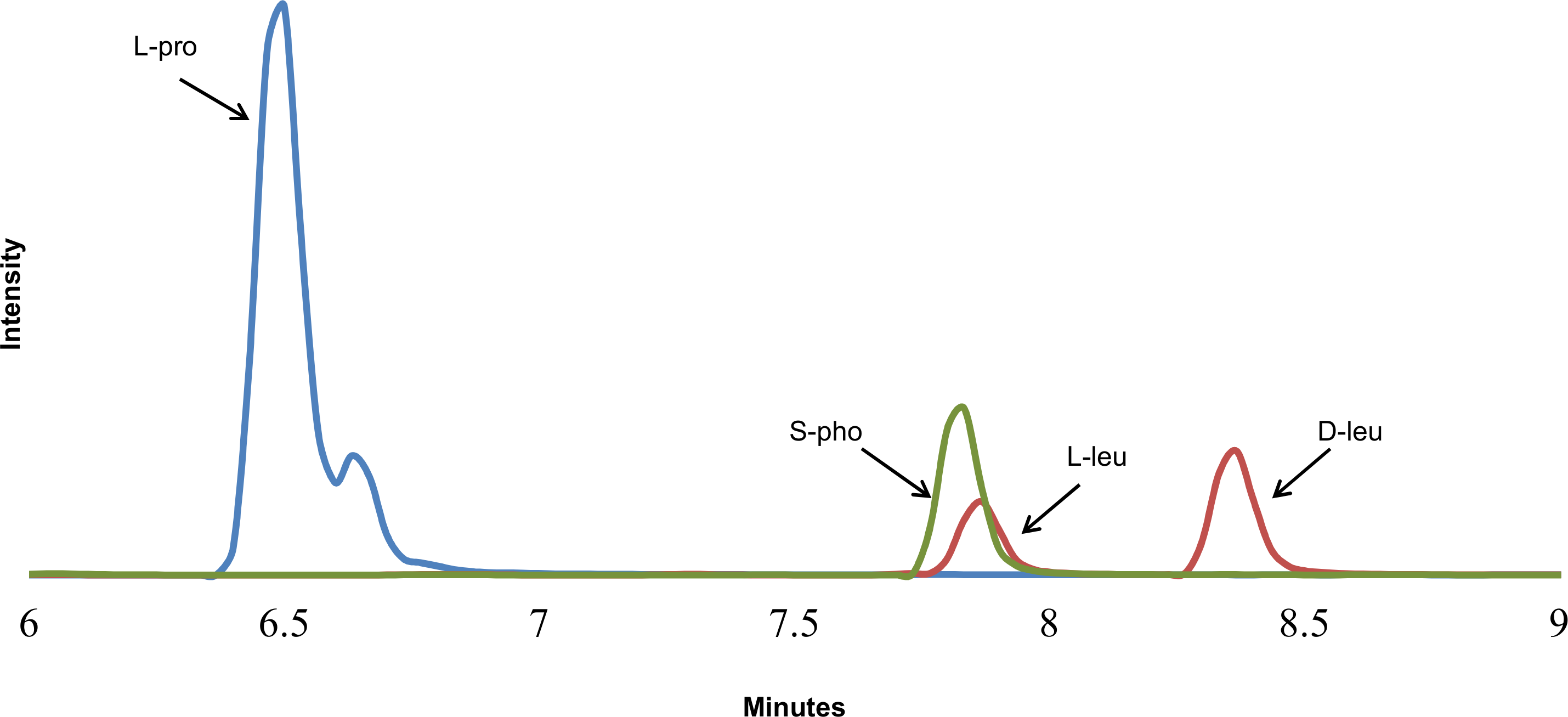

Supplement: Figure S2 — Overlaid selective ion trace chromatograms showing the C18 LC-MS results of the Marfey's analysis. Single ion trace detection of the derivatized amino acids: derivatized proline (blue), leucine (red), and phenylalaninol (green). (TIF) [file pone.0090124.s002.tif]

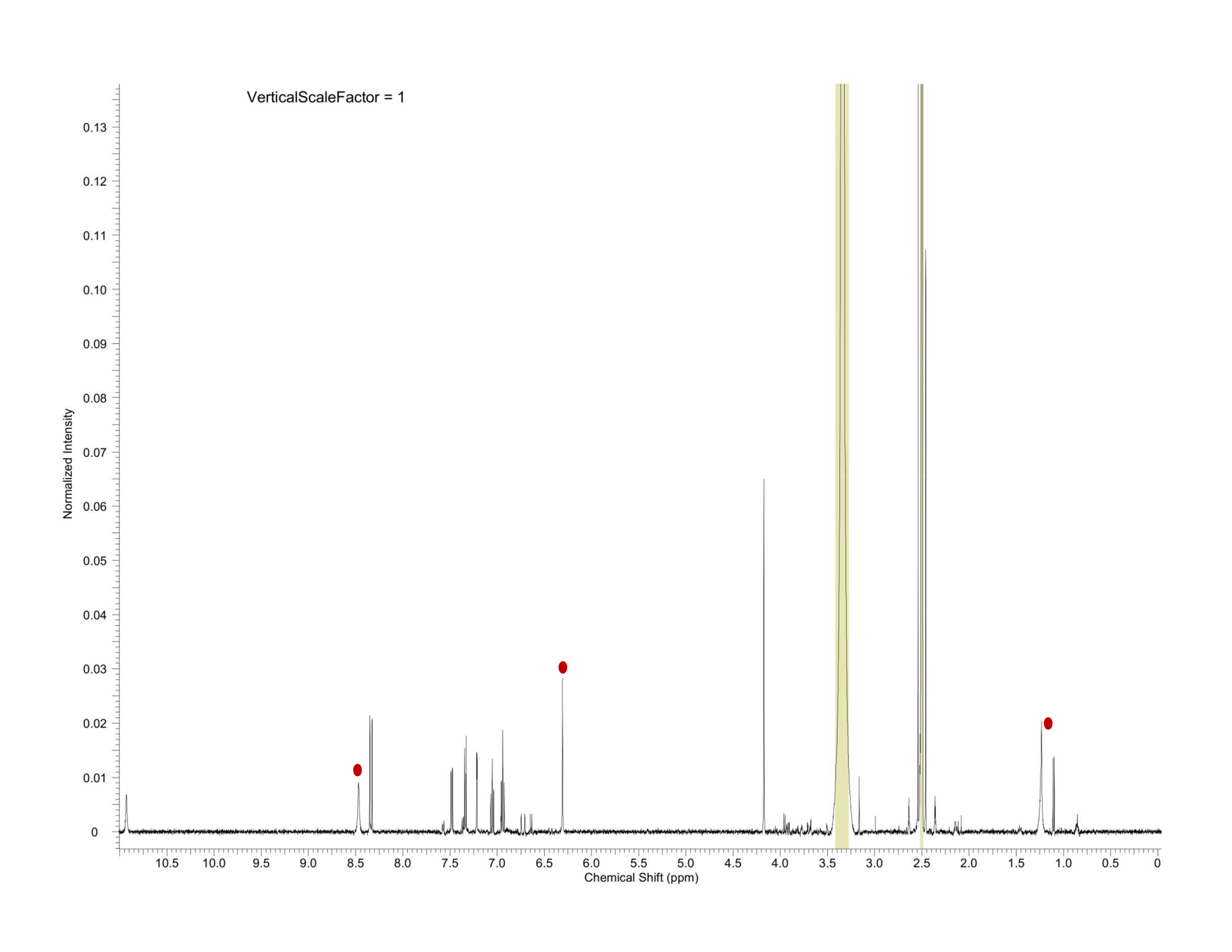

Supplement: Figure S3 — 1H (DMSO- d 6, 25°C) spectrum of compound 5. Signals resulting from impurities are marked with red dots. (TIF) [file pone.0090124.s003.tif]

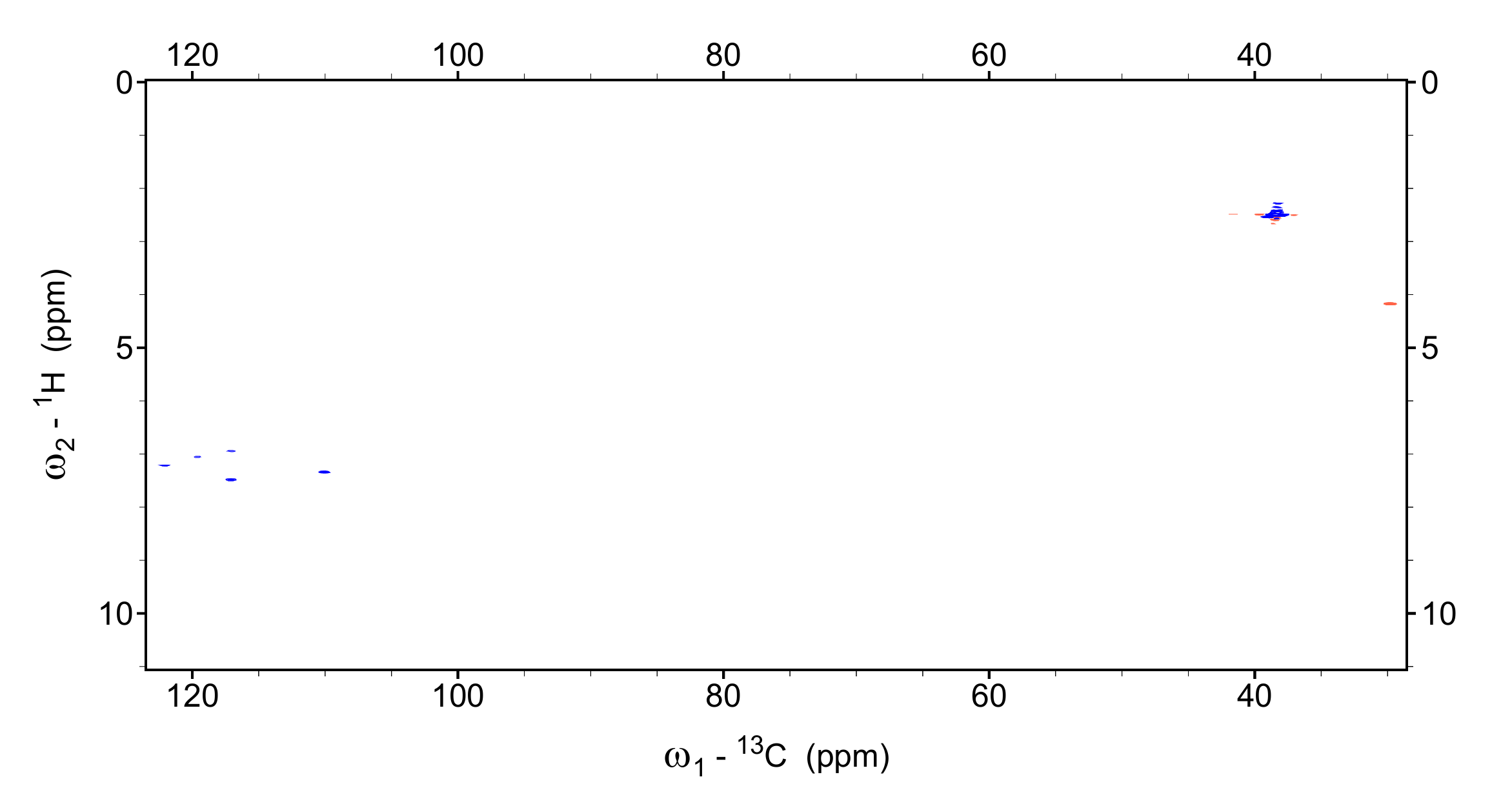

Supplement: Figure S4 — 1H-13C HSQC (DMSO- d 6, 25°C) spectrum of compound 5. CH groups are shown in blue, CH2 groups are in red. (TIF) [file pone.0090124.s004.tif]

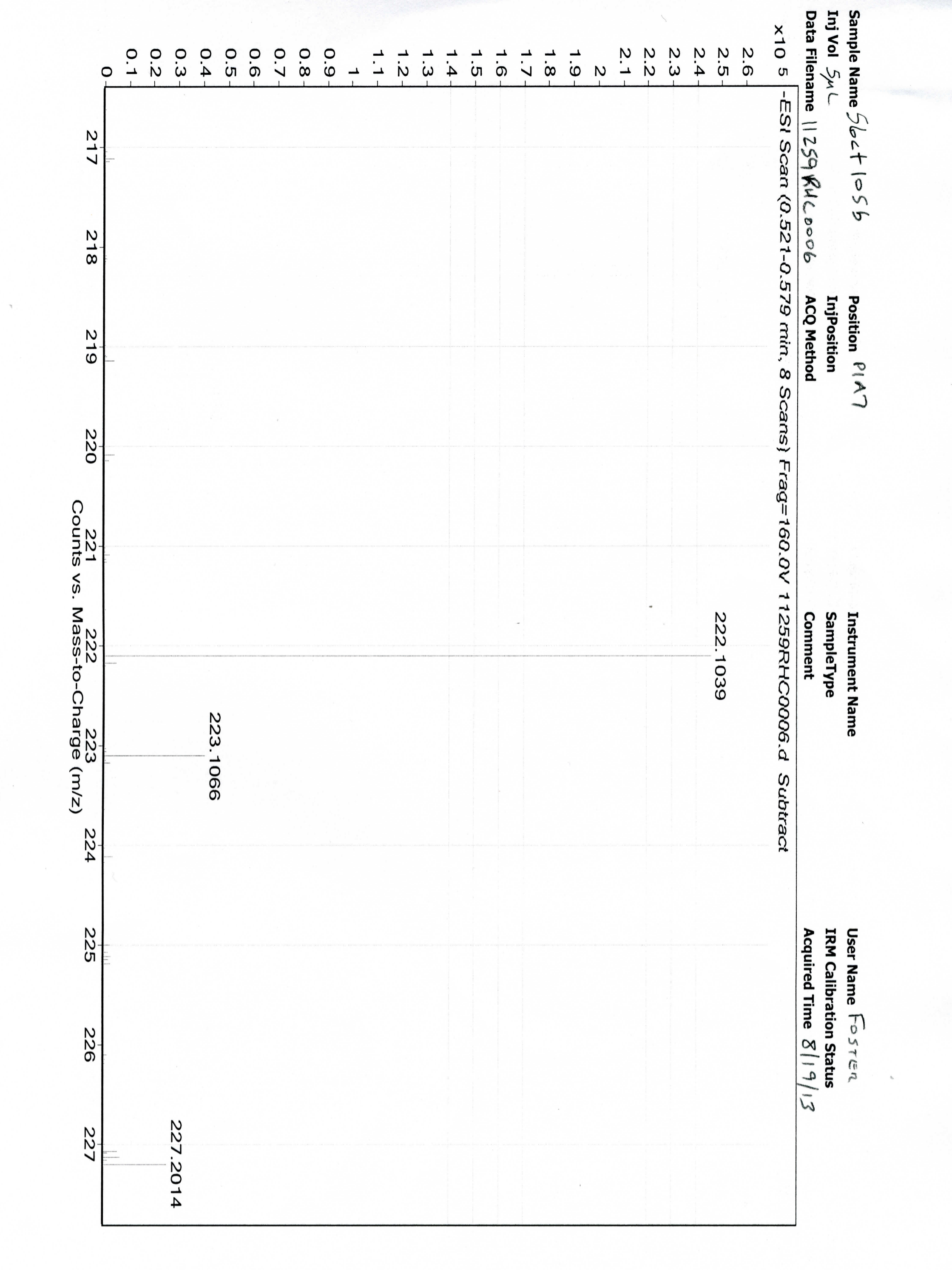

Supplement: Figure S6 — HRESI-MS (negative mode) data for compound 5. (TIF) [file pone.0090124.s006.tif]

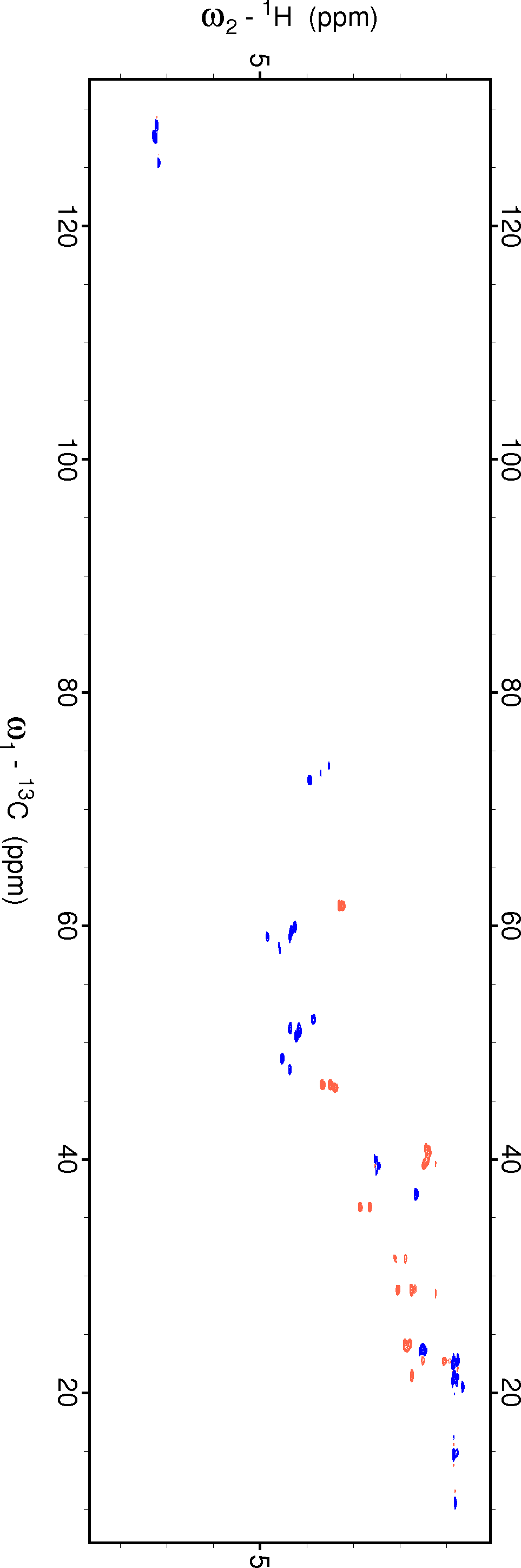

Supplement: Figure S9 — 1H-13C HSQC (DMSO- d 6, 25°C) spectrum of compound 6. CH groups are shown in blue, CH2 groups are in red. (TIF) [file pone.0090124.s009.tif]

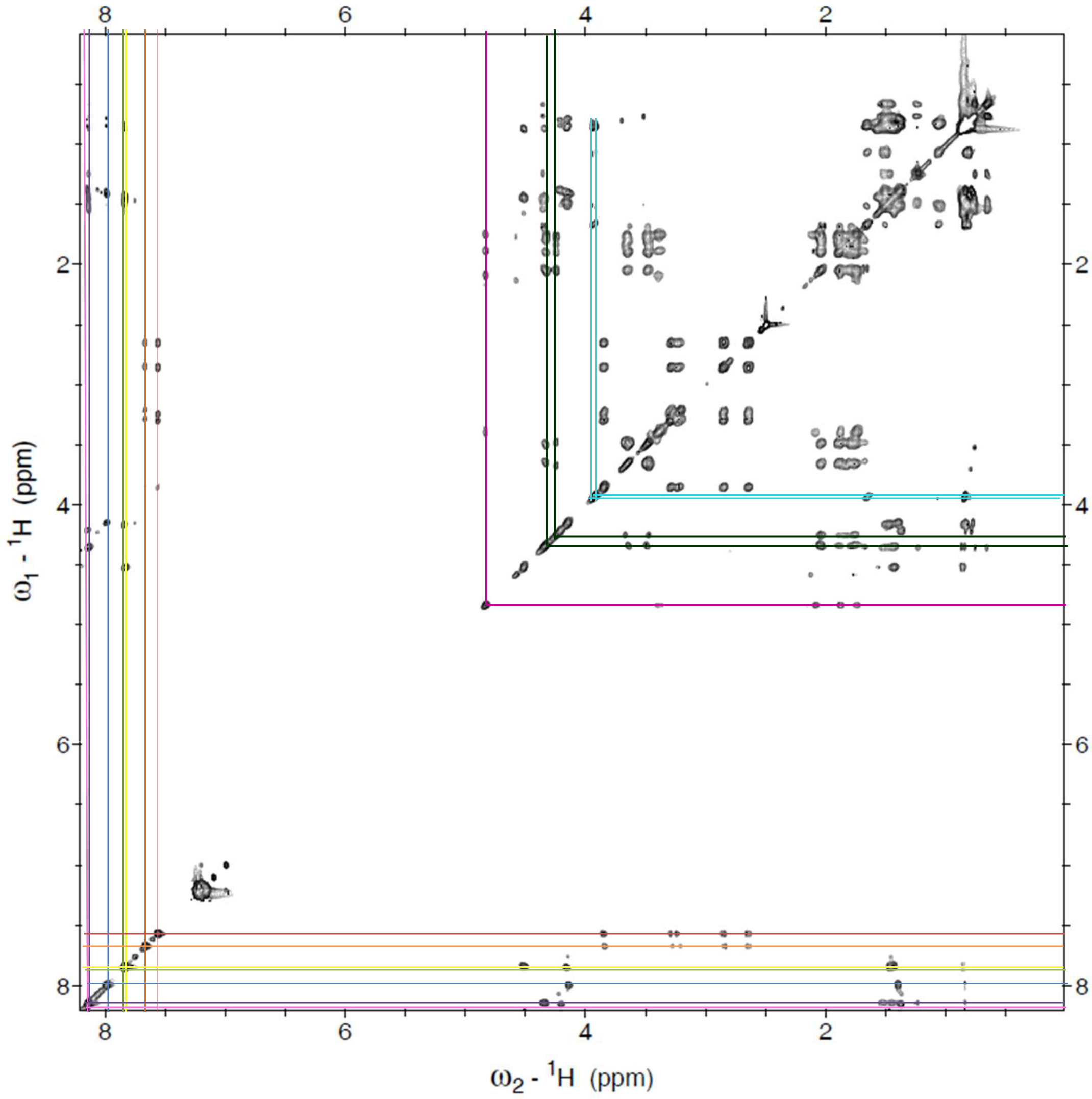

Supplement: Figure S11 — 1H-13C TOCSY (DMSO- d 6, 25°C) spectrum of compound 6. (TIF) [file pone.0090124.s011.tif]

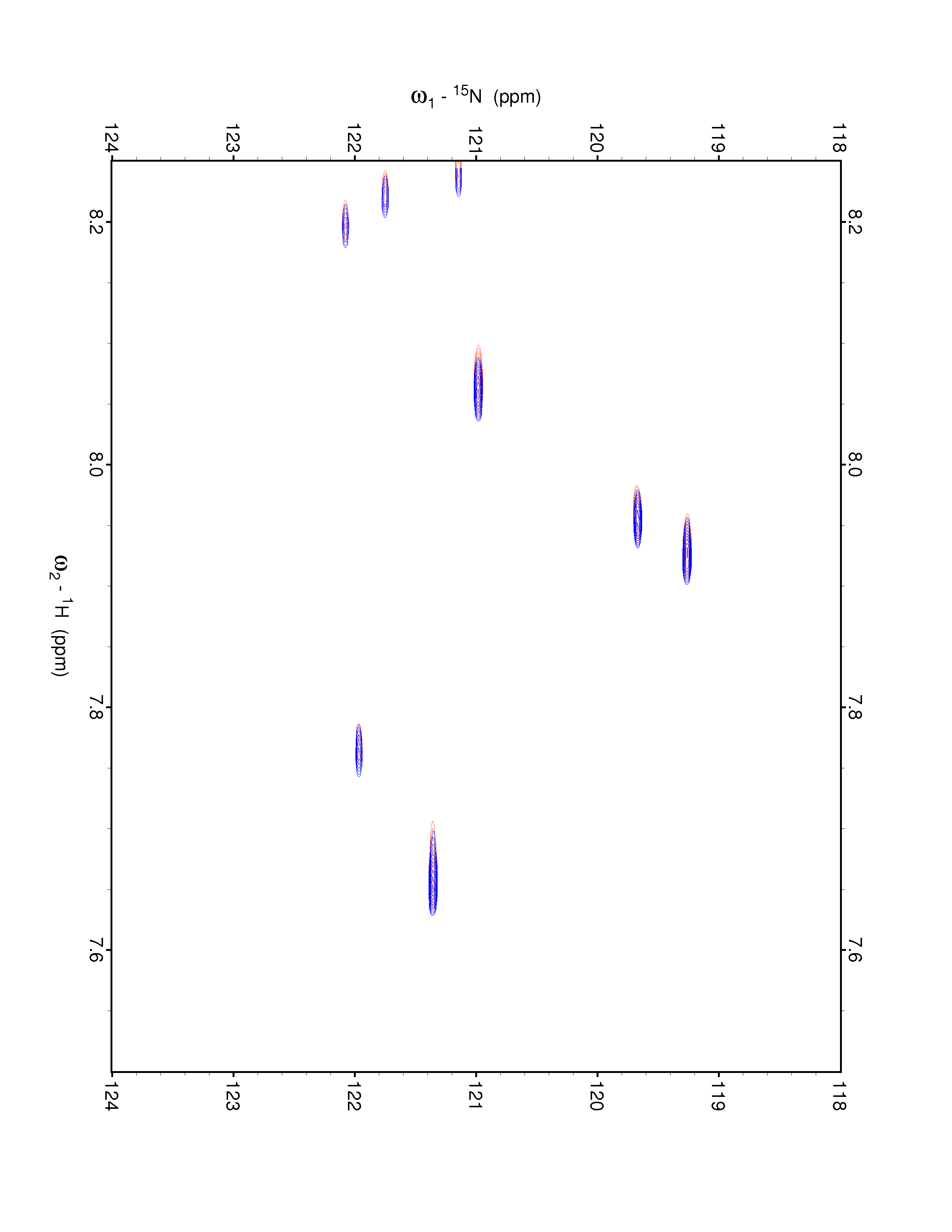

Supplement: Figure S14 — Overlaid 1H-14N HSCQ (DMSO- d 6, 25°C) spectra of compound 6. Overlay of two NMR experiments. Data from the first experiment, optimized to show NH signals only, are illustrated in red. Data from the second experiment, optimized to show both NH and NH2, are illustrated in blue. Data from both experiments show complete overlap indicating there are no secondary amines. (TIF) [file pone.0090124.s014.tif]

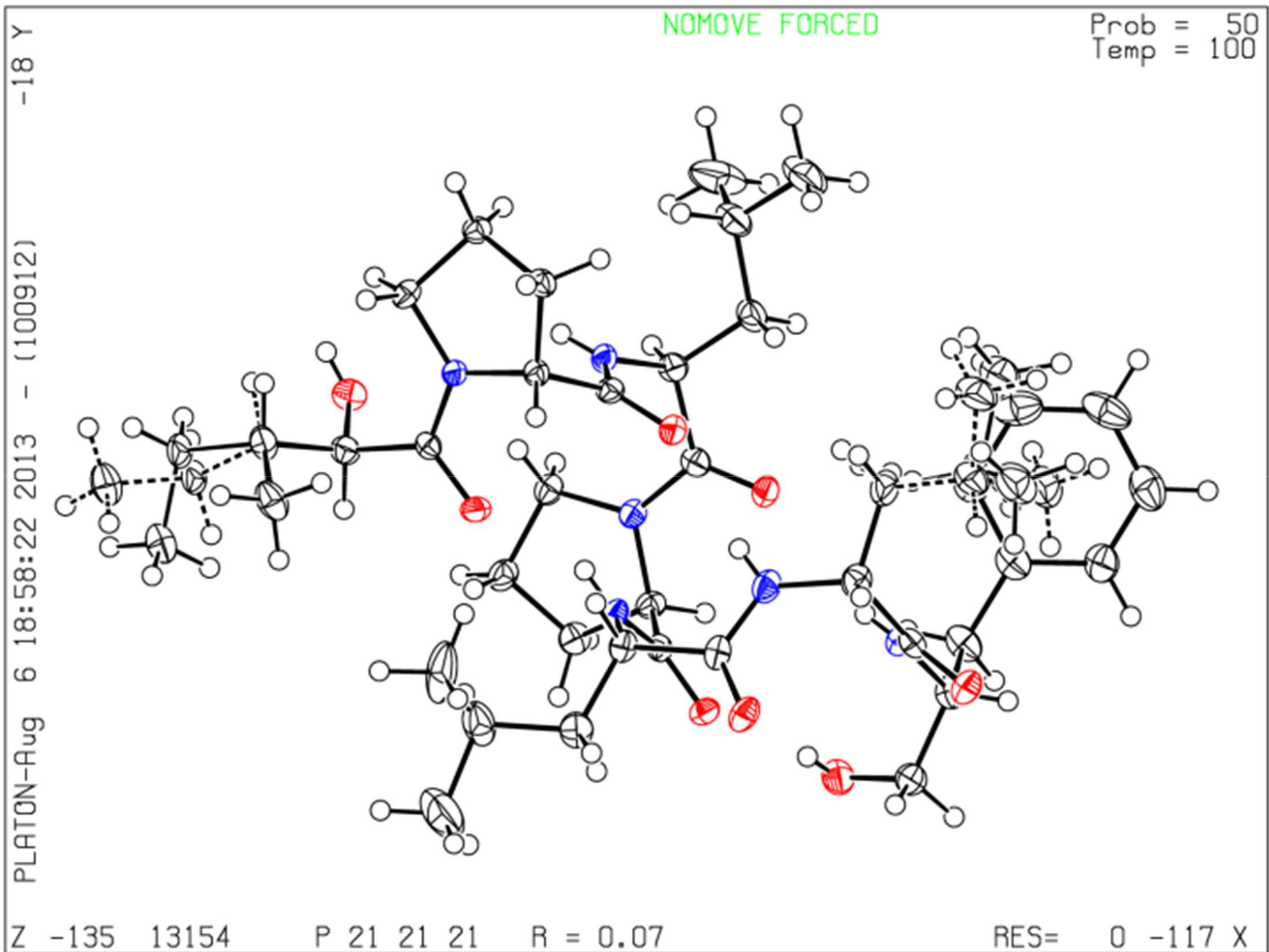

Supplement: Figure S15 — Thermal ellipsoid plot of compound 6. (TIF) [file pone.0090124.s015.tif]
