# Supplementary material for: Genomic and Metabolomic Insights into the Natural Product Biosynthetic Diversity of a Feral-Hog-Associated Brevibacillus laterosporus Strain
Source: PLoS One. 2014 Mar 3;9(3):e90124. doi: 10.1371/journal.pone.0090124 (PMC3940840; doi:10.1371/journal.pone.0090124)
Supplement: Table S1 — Crystal data and structure refinement data for compound 6. (DOCX) [file pone.0090124.s019.docx]

**Table S1**. Crystal data and structure refinement for **6**.

| Emperical formula | C_43_H_70_N_6_O_8_ |
| --- | --- |
| Formula weight | 799.05 |
| Crystal system | Orthorhombic |
| Space group | P2_1_2_1_2_1_ |
| Unit cell dimensions | a = 11.1021(10) Å α = 90^o^ |
|  | b = 14.9391(14) Å β = 90^o^ |
|  | c = 26.211(2) Å γ = 90^o^ |
| Volume | 4347.2(7) Å^3^ |
| Z,Zʹ | 4, 1 |
| Density (calculated) | 1.221 Mg/m^3^ |
| Wavelength | 0.71073 Å |
| Temperature | 100(2) K |
| F(000) | 1736 |
| Absorption coefficient | 0.084 mm^-1^ |
| Absorption correction | Semi-empirical from equivalents |
| Max. and min. transmission | 0.990 and 0.964 |
| Theta range for data collection | 1.554 to 28.344^o^ |
| Reflections collected | 40661 |
| Independent reflections | 10800 [R(int) = 0.0586] |
| Data / restraints / parameters | 10800 / 118 / 573 |
| wR(F^2^ all data) | wR2 = 0.1925 |
| R(F obsd data) | R1 = 0.0707 |
| Goodness-of-fit on F^2^ | 1.011 |
| Observed data [I>2σ(I)] | 9029 |
| Absolute structure parameter | 1.2(6) |
| Largest and mean shift / s.u. | 0.012 and 0.000 |
| Largest diff. peak and hole | 0.315 and -0.374 |

wR2 = {Σ [w (F_o_^2^-F_c_^2^)^2^] / Σ [ w (F_o_^2^)^2^]}^1/2^

R1 = Σ ||F_o_| - |F_c_|| / Σ |F_o_|
